# The economic impact of Long-Acting Contraceptives (LARCs) on public health

**DOI:** 10.1016/j.clinsp.2025.100598

**Published:** 2025-02-19

**Authors:** Ana Claudia Marcelino, Paula da Cunha Pereira, Luis Bahamondes

**Affiliations:** a Department of Obstetrics and Gynecology, Faculdade de Medicina da Universidade Estadual de Campinas, Campinas, SP, Brazil; b Center for Research in Reproductive Health of Campinas (CEMICAMP), Campinas, SP, Brazil

**Keywords:** Brazil, Contraception, Economics

## Abstract

The incorporation of long-acting reversible contraceptives (LARCs) into reproductive health strategies is crucial for enhancing access to effective contraception and reducing unplanned pregnancies (UPs). Evidence shows that even a modest shift from short-acting methods to LARCs can yield significant cost savings and improve health outcomes, both for individuals and public health systems. By facilitating single-visit placements and expanding the training of multidisciplinary teams, including nurses, healthcare providers can increase access to these effective contraceptive options.

## Highlights

• The use of LARCs reduces unplanned pregnancies.
• Single-visit placements of LARCs increase access and uptake.
• Train and allowed nurses on the placement of LARCs is important to women access and uptake.
• Expand the knowledge of non-contraceptive benefits of LARCs is important among providers and users.

## Introduction

The United Nations Target 3.7 of the 2030 Agenda for Sustainable Development Goals aims to ensure universal access to sexual and reproductive health services, including modern contraceptive methods and education.^1^ The shift from “family planning” to “reproductive planning” reflects diverse family structures today, emphasizing the importance of individual choice in making reproductive decisions. Key indicators for this target include the proportion of women (aged 15–49) using modern contraceptives and the adolescent birth rate per 1000 women.^1^

Regarding the growth projection of the global population, it is estimated to reach approximately 8.79 billion people by the year 2100.^2^ In terms of the Agenda for Sustainable Development Goals, reducing this percentage could lower global population growth by up to 6.29 billion people.^2^ This reduction would significantly lessen the strain on public health systems worldwide, resulting in substantial savings on costs related to prenatal care, childbirth, and infant care,^3^^,^^4^ particularly in socioeconomically vulnerable regions. Furthermore, a smaller planetary population can mean lower carbon emissions and a reduced likelihood of transgressing planetary boundaries, i.e., reducing the risk of human interventions destabilizing the Earth system.^5^^,^^6^

This opinion article explores how Long-Acting Reversible Contraceptive methods (LARCs) can contribute to achieving these goals, lowering public health costs, preventing unplanned pregnancies and their consequences, and reducing the planetary population and its consequent global economic impact.

LARCs are a category of contraceptive methods designed to provide effective pregnancy prevention for extended periods, typically ranging from three years to over a decade, without requiring daily attention.^7^ The two main types of LARCs are Intrauterine Devices (IUDs) and subdermal implants. IUDs can be either copper-bearing, which creates a hostile environment for sperm, or hormonal, which releases Levonorgestrel (LNG) to thicken cervical mucus and prevent ovulation. Subdermal implants are small rods placed under the skin of the arm that release hormones to inhibit ovulation.^8^ LARCs are highly effective, with failure rates of less than 1/100 Women-Years (W-Y) with typical use, making them one of the most reliable forms of contraception available even for adolescents and nulligravidas.^9^ They are also reversible, meaning that fertility returns quickly upon removal, making them an ideal choice for women who want long-term contraceptive protection without the need for frequent intervention.^10^

In Brazil, family planning is a legal right guaranteed by Federal Law 9.263/96, which assures citizens of access to reproductive health services.^11^ Through the Unified Health System (SUS), the government provides free contraceptives such as condoms, combined and progestin oral contraceptives, injectables, and Intrauterine Devices (IUDs). Family planning plays a critical role in reducing Unplanned Pregnancies (UPs), as well as maternal and infant morbidity and mortality, by allowing women to space pregnancies, which is particularly important for young women who face higher health risks and complications associated with early pregnancies.^12^ Access to LARCs has reduced costs and health risks.^3^^,^^4^^,^^12^^,^^13^

A study estimated that the annual cost of Brazil's UPs was almost R$4.1 billion, with most expenses related to antenatal care, delivery, and postnatal complications. Direct birth costs alone account for approximately R$1.22 billion, while infant complications and hospital readmissions represent a substantial proportion of these costs, totaling R$2.84 billion.^3^ Another study about UP costs, in Sweden, estimated that a 5% shift from non-LARCs to LARCs methods would result in over 3500 fewer UPs annually, leading to savings of nearly €7.7 million.^4^ In Norway, the total costs of UPs in young women were estimated to be 164 million Norwegian Kroner (NOK), of which 81.7% was thought to be due to imperfect contraceptive adherence. It was projected that a 5% increase in LARC uptake would lead to cost savings of NOK 7.2 million in this group.^14^ In adolescents, a systematic review confirmed the cost-effectiveness of introducing LARCs and preventing Ups.^15^

An extensive review of users of our family planning service revealed that, from 1980 to 2012, approximately 20,000 women started using LARCs, which in the last decade have contributed to preventing 37 to 60 maternal deaths, 315 to 424 infant deaths, 634 to 853 cases of combined maternal and infant morbidity and mortality, and 1056 to 1412 unsafe abortions.^16^ In Brazil, the authors know that the use of LARCs is cost-effective when compared to Combined Oral Contraceptives (COCs) and can save about $65 billion for the LNG 52 mg IUD, $302 billion for the subdermal implant, and up to $422 billion for the copper IUD in the five years following its implementation.^17^ There is also positive evidence of the cost-effectiveness of implant insertion as a public health measure in large countries such as India and Brazil.^18^^,^^19^

It is known that contraceptive use is higher when more contraceptive methods are available to a large part of the population,^20^ and is better than solutions such as cash transfer interventions.^21^ In 2020, the estimated use of LARCs was 19.4% globally, while the use of Short-Acting methods (SARCs) was 45%.^22^ In contrast, the worldwide use of contraceptive implants in 2020 was 2.6%.^23^ In Latin America and the Caribbean, there are recommendations to consider LARCs as a primary option for decreasing UPs and adolescent pregnancy,^24^ and its use is still not uniform in the region. Recent evidence shows that the satisfied demand for the use of modern contraceptives in Latin America and the Caribbean is directly related to the educational and socioeconomic level of women, and indirectly related to the level of gender inequality.^25^

Facilitated access to LARC options must be a goal since their single-visit placements represent an effective strategy, resulting in overall very low UP rates, and practicing the extended use of LARCs, as recommended by the US Society of Family Planning.^26^ This approach has the potential to reduce not only UPs but also the costs and barriers faced by women and the healthcare system.^13^ The insertion of LARCs is an outpatient safe reversible procedure and less invasive than surgical methods. Consequently, IUD insertion in the immediate postpartum period is recognized as an effective strategy to enhance coverage and access to contraception.^27^ Although there may be a slightly higher expulsion rate associated with IUDs inserted immediately after delivery, the benefits of immediate access to reliable contraception outweigh this inconvenience.^28^

Moreover, implementing a training program for a multidisciplinary team can significantly improve access to LARCs, allowing the range of providers capable of offering these methods to expand beyond just gynecologists. Evidence suggests that clinical outcomes remain consistent regardless of whether the insertion is performed by nurses, residents, or physicians, as well as irrespective of the age and parity of users. This indicates that healthcare services, especially in areas facing a shortage of physicians, should consider training nurses in IUD insertion to facilitate access to effective contraceptive methods.^29^^,^^30^

LARCs not only reduce the costs for public health, reducing the number of UPs and medical visits but also significantly lower general costs for women, as they no longer need to frequently travel for contraceptive replenishment.^13^ LARCs are associated with relatively few side effects compared to other contraceptive methods,^31^ which benefit women's health, but also lessen the financial burden on healthcare systems, minimizing costs associated with treating several conditions.^32^

In addition to their contraceptive efficacy, hormonal LARCs provide several non-contraceptive benefits that can further reduce healthcare costs. Many hormonal IUDs and subdermal implants can alleviate Heavy Menstrual Bleeding (HMB), decrease the incidence of anemia, and reduce the need for surgeries related to reproductive health issues, as well as alleviate symptoms of conditions such as endometriosis and adenomyosis.^33^^,^^34^ By addressing these health issues, LARCs not only improve women's quality of life but also lead to significant savings on hygiene products and medical interventions.^35^ The use of disposable pads also imposes the need to buy them, which is not always possible. Menstrual poverty is a global problem,^38^ and in Brazil, the scenario in relation to menstrual rights is worrying, marked by historical inequalities in terms of gender, race, region, and social class, aggravated in times of health and economic crises.^36^

The use of LARCs could be at the heart of the solution to the biggest public health problem we will face in the coming years: climate change. By aiding conscious reproductive planning, reducing gender inequalities, promoting sexual and reproductive education, and guaranteeing basic rights for all, we improve the health of humanity, but also planetary health. The impacts of human activity on air and water pollution, biodiversity loss, and climate change are notorious,^5^^,^^6^ especially in terms of production and economic growth. LARCs can help to put a ceiling on the harmful growth of humanity, but not without consequences: Current economic models need the growth of the economically active population to manage social security programs and to finance public health systems themselves.^37^ Among the potential changes that LARCs bring to the economy, in the long term, it could reduce human intervention on Earth.

## Conclusion

The incorporation of LARCs into reproductive health strategies is crucial for enhancing access to effective contraception and reducing UPs. Evidence shows that even a modest shift from short-acting methods to LARCs can yield significant cost savings and improve health outcomes, both for individuals and public health systems. By facilitating single-visit placements and expanding the training of multidisciplinary teams, including nurses, healthcare providers can increase access to these effective contraceptive options. Additionally, the non-contraceptive benefits of LARCs further emphasize their role in improving women's health. Ultimately, prioritizing LARC usage aligns with global health goals and empowers women to make informed reproductive choices. Also, there is robust evidence of the cost-effectiveness of the universal introduction of hormonal IUDs and contraceptive implants in public health. An aging population and the consequent pressure on health systems, social security programs, and the workforce, at different rates in different regions of the planet, could lead to economic and geopolitical instability. Therefore, it would be necessary to think about the use of LARCs.

## Declaration of competing interest

The authors declare no conflicts of interest.

## References

[1] United Nations. 2023. Goal 3.7: ensure universal access to sexual and reproductive health services ‒ United Nations Sustainable Development [Internet]. [cited 2024 Oct 14]. Available from: https://www.un.org/sustainabledevelopment/health/.

[2] Vollset S.E., Goren E., Yuan C.W., Cao J., Smith A.E., Hsiao T. (2020). Fertility, mortality, migration, and population scenarios for 195 countries and territories from 2017 to 2100: a forecasting analysis for the Global Burden of Disease Study. Lancet.

[3] Le H.H., Connolly M.P., Bahamondes L., Cecatti J.G., Yu J., Hu H.X. (2014). The burden of unintended pregnancies in Brazil: a social and public health system cost analysis. Int J Women Health.

[4] Engstrand S., Kopp Kallner H. (2018). Cost of unintended pregnancy in Sweden – a possibility to lower costs by increasing LARC usage. Contraception.

[5] Shi A. (2003). The impact of population pressure on global carbon dioxide emissions, 1975–1996: evidence from pooled cross-country data. Ecological Economics.

[6] Steffen W., Richardson K., Rockström J., Cornell S.E., Fetzer I., Bennett E.M. (2015). Sustainability. Planetary boundaries: guiding human development on a changing planet. Science (1979).

[7] Amaral G., Foster D.G., Biggs M.A., Jasik C.B., Judd S., Brindis C.D. (2007). Public savings from the prevention of unintended pregnancy: a cost analysis of family planning services in California. Health Serv Res.

[8] Stoddard A., McNicholas C., Peipert J.F. (2011). Efficacy and safety of long-acting reversible contraception. Drugs.

[9] Laporte M., Marcelino A.C., Pereira P.D.C., Espejo-Arce X., Juliato C.T., Bahamondes L. (2024). Uptake, discontinuation, and continuation rate of long-acting contraceptive methods when offered at no cost in Campinas. Brazil. Contraception..

[10] Girum T., Wasie A. (2018). Return of fertility after discontinuation of contraception: a systematic review and meta-analysis. Contracept Reprod Med.

[11] Lei No 9.263 (1996). Regula o § 7o do art. 226 da Constituição Federal, que trata do planejamento familiar, estabelece penalidades e dá outras providências. Diário Oficial da União [Internet].

[12] de Azevedo WF, MB Diniz, Fonseca E.S.V.B., de Azevedo LMR, Evangelista C.B (2015). Complications in adolescent pregnancy: systematic review of the literature. Einstein (Sao Paulo).

[13] Manhiça S.I., Bahamondes L., Laporte M., Anjos F., Viscola M., Garcia E. (2023). Single-visit long-acting reversible contraception provision and pregnancy rates within 3 months. Int J Gynaecol Obstet.

[14] Henry N., Schlueter M., Lowin J., Lekander I., Filonenko A., Trussell J. (2015). Cost of unintended pregnancy in Norway: a role for long-acting reversible contraception. J Fam Plann Reprod Health Care.

[15] Marmett B., Guaranha D.D.F.K., de Carvalho AF, Reis J.M., de Souza CLE, Dalcin T.C. (2024). Cost savings and effectiveness of long-acting reversible contraception (LARC) on the prevention of pregnancy in adolescents: a systematic review. J Pediatr Adolesc Gynecol.

[16] Bahamondes L., Bottura B.F., Bahamondes M.V., Gonçalves M.P., Correia V.M., Espejo-Arce X. (2014). Estimated disability-adjusted life years averted by long-term provision of long-acting contraceptive methods in a Brazilian clinic. Hum Reprod.

[17] Farah D., de Mohraes Andrade T.R., Sansone D., Batista Castello Girão M.J., Fonseca M.C.M (2022). A cost effectiveness model of long-acting reversible contraceptive methods in the Brazilian national health system. Am J Prev Med.

[18] Joshi B., Moray K.V., Sachin O., Chaurasia H., Begum S. (2021). Cost Effectiveness of Introducing Etonorgestrel Contraceptive Implant into India's Current Family Welfare Programme. Appl Health Econ Health Policy.

[19] Lopes da Silva Filho A., Bueno L.P., Ramires Y., Lino L.M.C (2024). Etonogestrel-releasing subdermal contraceptive implant: budget impact analysis based on the Brazilian private healthcare system. PLoS One.

[20] Ross J., Stover J. (2013). Use of modern contraception increases when more methods become available: analysis of evidence from 1982 to 2009. Glob Health Sci Pract.

[21] Kneale D., Kjaersgaard A., de Melo M., Joaquim Picardo J., Griffin S., French R.S. (2023).

[22] (2022).

[23] Bahamondes L., Villarroel C., Frías Guzmán N., Oizerovich S., Velázquez-Ramírez N., Monteiro I (2018). The use of long-acting reversible contraceptives in Latin America and the Caribbean: current landscape and recommendations. Hum Reprod Open.

[24] Moreira L.R., Blumenberg C., Caicedo Velasquez B.E., Ewerling F., Balandrán A., Vidaletti L.P. (2023). The role of gender inequality and health expenditure on the coverage of demand for family planning satisfied by modern contraceptives: a multilevel analysis of cross-sectional studies in 14 LAC countries. Lancet Reg Health Am.

[25] Dethier D., Qasba N., Kaneshiro B. (2022). Society of Family Planning clinical recommendation: extended use of long-acting reversible contraception. Contraception.

[26] (2016). American College of Obstetricians and Gynecologists’ Committee on Obstetric Practice. Committee Opinion No. 670: immediate Postpartum Long-Acting Reversible Contraception. Obstet Gynecol.

[27] Herculano T.B., Surita F.G., Juliato C.R.T., Rehder P.M. (2022). Comparison between two methods of the immediate post-placental insertion of copper intrauterine device in vaginal birth-a protocol for a randomized clinical trial. Trials.

[28] Laporte M., Becerra A., Castro L., Jr Veiga N, Espejo-Arce X., Bahamondes L (2021). Evaluation of clinical performance when intrauterine devices are inserted by different categories of healthcare professional. Int J Gynaecol Obstet.

[29] Millogo T., Chomi E., Kouanda S., Ali M. (2023). Getting Up to Date with What Works: a Systematic Review on the Effectiveness and Safety of Task Sharing of Modern Methods in Family Planning Services. Biomed Res Int.

[30] Lima A.C.S., Martins L.C.G., Lopes M.V.O., Araújo T.L., Lima F.E.T., Aquino P.S. (2017). Influence of hormonal contraceptives and the occurrence of stroke: integrative review. Rev Bras Enferm.

[31] Strilciuc S, Grad DA, Radu C, Chira D, Stan A, Ungureanu M, Gheorghe A, Muresanu FD (2021). The economic burden of stroke: a systematic review of cost of illness studies. J Med Life.

[32] Bahamondes L., Valeria Bahamondes M., Shulman L.P (2015). Non-contraceptive benefits of hormonal and intrauterine reversible contraceptive methods. Hum Reprod Update.

[33] Viscola M, Marcelino AC, da C., Pereira P, Monteiro I, Espejo-Arce X, Bahamondes L. (2014). Improvement of laboratory markers of anaemia in the treatment of heavy menstrual bleeding with a 19.5-mg intrauterine device: a pilot study. Eur J Contrac Reprod Health Care.

[34] Regional Health-Americas TL (2022). Menstrual health: a neglected public health problem. Lancet Reg Health Am.

[35] Jaafar H., Ismail S.Y., Azzeri A. (2023). Period Poverty: a Neglected Public Health Issue. Korean J Fam Med.

[36] (2021). https://www.unicef.org/brazil/relatorios/pobreza-menstrual-no-brasil-desigualdade-e-violacoes-de-direitos.

[37] Iyer H.S., DeVille N.V., Stoddard O., Cole J., Myers S.S., Li H. (2021). Sustaining planetary health through systems thinking: public health's critical role. SSM Popul Health.

[38] GBD 2021 Fertility and Forecasting Collaborators (2024). Global fertility in 204 countries and territories, 1950-2021, with forecasts to 2100: a comprehensive demographic analysis for the Global Burden of Disease Study 2021. Lancet.

